# Mapping Asbestos-Cement Roofing with Hyperspectral Remote Sensing over a Large Mountain Region of the Italian Western Alps

**DOI:** 10.3390/s140915900

**Published:** 2014-08-27

**Authors:** Federico Frassy, Gabriele Candiani, Marco Rusmini, Pieralberto Maianti, Andrea Marchesi, Francesco Rota Nodari, Giorgio Dalla Via, Carlo Albonico, Marco Gianinetto

**Affiliations:** 1 Laboratory of Remote Sensing (L@RS), Politecnico di Milano—Department of Architecture, Built Environment and Construction Engineering (ABC), Via Ponzio 31, Milano 20133, Italy; E-Mails: federico.frassy@polimi.it (F.F.); pieralberto.maianti@polimi.it (P.M.); andrea.marchesi@polimi.it (A.M.); francesco.rotanodari@polimi.it (F.R.N.); giorgio.dallavia@polimi.it (G.D.V.); 2 Optical Remote Sensing Group, IREA-CNR, Via Bassini 15, Milano 20133, Italy; E-Mail: candiani.g@irea.cnr.it; 3 ERM Italia S.p.A., Via San Gregorio 38, Milano 20124, Italy; E-Mail: marco.rusmini@erm.com; 4 ARPA Valle d'Aosta, Loc. Grande Charrière, 44, Saint-Christophe (AO) 11020, Italy; E-Mail: c.albonico@arpa.vda.it

**Keywords:** asbestos-cement, hyperspectral, remote sensing, mapping

## Abstract

The World Health Organization estimates that 100 thousand people in the world die every year from asbestos-related cancers and more than 300 thousand European citizens are expected to die from asbestos-related mesothelioma by 2030. Both the European and the Italian legislations have banned the manufacture, importation, processing and distribution in commerce of asbestos-containing products and have recommended action plans for the safe removal of asbestos from public and private buildings. This paper describes the quantitative mapping of asbestos-cement covers over a large mountainous region of Italian Western Alps using the Multispectral Infrared and Visible Imaging Spectrometer sensor. A very large data set made up of 61 airborne transect strips covering 3263 km^2^ were processed to support the identification of buildings with asbestos-cement roofing, promoted by the Valle d'Aosta Autonomous Region with the support of the Regional Environmental Protection Agency. Results showed an overall mapping accuracy of 80%, in terms of asbestos-cement surface detected. The influence of topography on the classification's accuracy suggested that even in high relief landscapes, the spatial resolution of data is the major source of errors and the smaller asbestos-cement covers were not detected or misclassified.

## 1. Introduction

The hazards of asbestos dust on human health have been documented since 1924 [1]. Nowadays, several studies have confirmed that exposure to asbestos fibers bestows a long-term risk of developing pleural mesothelioma [2], lung cancer [3], cancer of the larynx [4], ovarian cancer [5] and asbestosis [6], even when people are exposed to very low levels of asbestos fibers and after the cessation of exposure. Exposure to asbestos fibers scattered in the air occurs through inhalation in production sites, nearby factories handling asbestos, inside the homes of asbestos factory workers or in buildings containing friable or deteriorated asbestos materials. According to some recent estimates by the World Health Organization, about 125 million people in the world are exposed to asbestos at the workplace, more than 100 thousand people die every year from asbestos-related cancers whereof several thousand deaths can be ascribed to asbestos exposure at home [7]. The same organization estimated about 20–30 thousand cases of asbestos-related diseases per year in the European Union, asserting that the phenomenon has not yet reached its peak and more than 300 thousand European citizens are expected to die from mesothelioma by 2030. Asbestos has been indeed used for decades in Europe in the construction industry for thermal and fire insulation. Moreover, the fibers were often mixed with cement (asbestos-cement) into prefabricated elements, corrugated roofing sheets or tiles.

Although asbestos is today banned in more than 50 countries worldwide, it is still mined and used in many countries. The world leaders in asbestos production include the Russian Federation, China, the Republic of Kazakhstan, the Federative Republic of Brazil, Canada, the Republic of Zimbabwe and the Republic of Colombia. The largest consumers are China and the Republic of India, but asbestos is also used in most of Asia, Eastern Europe, Latin America and Africa. In the United States, the Toxic Substances Control Act issued by the US Environmental Protection Agency (1989) banned the manufacture, import, processing and distribution in commerce of most asbestos-containing products [8]. However, few years later (1991) most of the original ban was overturned and only certain asbestos-containing products such as corrugated paper, rollboard, commercial paper, specialty paper or flooring felt, as well as the use of asbestos in products that have not historically contained it, are still banned. Thus, several asbestos-containing products used in building construction, such as cement corrugated/flat sheets, roofing felts or roof coatings, are allowed. In contrast, the European Union restricted the marketing and use of products containing asbestos since 1976, with the Council Directive 76/769/CEE [9], and then banned asbestos at all in 1999, with the Commission Directive 99/77/CEE [10].

In this context, Italy completely banned the production and use of asbestos in 1992 with the Law 257/92 [11] and provided guidelines to map areas containing asbestos fibers with the National Decree (101/2003) [12], in accordance with the European recommendations to support action plans for the safe removal of asbestos from public and private buildings. The recent sentences of the lower (2012) and the appeal (2013) courts of Turin which condemned an industrialist of Eternit, the Swiss factory of asbestos cement having in the past a main production site in NW-Italy (Casale Monferrato) have recently renewed the interest on asbestos-related diseases in Europe. The trial is the biggest of its kind against a multinational for asbestos-related deaths.

In the past, remote sensing technologies were tested for the detection of asbestos-containing materials in buildings, in particular asbestos-cement roofing. Several research activities were carried out using the Multispectral Infrared and Visible Imaging Spectrometer (MIVIS) for mapping asbestos-cement roofs and for monitoring its deterioration status [13–20]. However, almost all past researches were limited to small test sites, mainly in urban or industrial areas and with rather flat topography, while systematic mapping over large areas was carried out only in a few cases [17,19]. This paper describes the mapping of asbestos-cement roofing with the MIVIS sensor over a large mountain region of Italian Western Alps for supporting decision making and action.

## 2. Methods

### 2.1. Study Area

**Figure 1.**
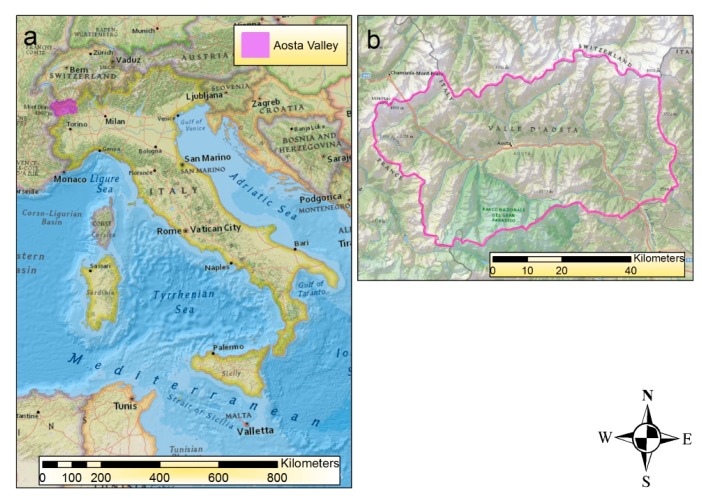
Overview of the study area. (**a**) Italy; (**b**) Valle d'Aosta Autonomous Region (North-West Italy).

Our study area was the Valle d'Aosta Autonomous Region (RAVA, Figure 1), the smallest Italian Region (3263 km^2^) located in the North-West of Italy, next to the border with France and Switzerland.

The region is a mountainous area and includes some of the highest mountains of the Alps: Mont Blanc (4810 m), Matterhorn (4478 m), Monte Rosa Massif (4634 m) and Gran Paradiso Massif (4061 m). The main central valley starts from Pont-Saint-Martin (312 m) and ends at Courmayeur (1224 m), passing through the city of Aosta (583 m) which is the main urban center.

### 2.2. Data

**Figure 2.**
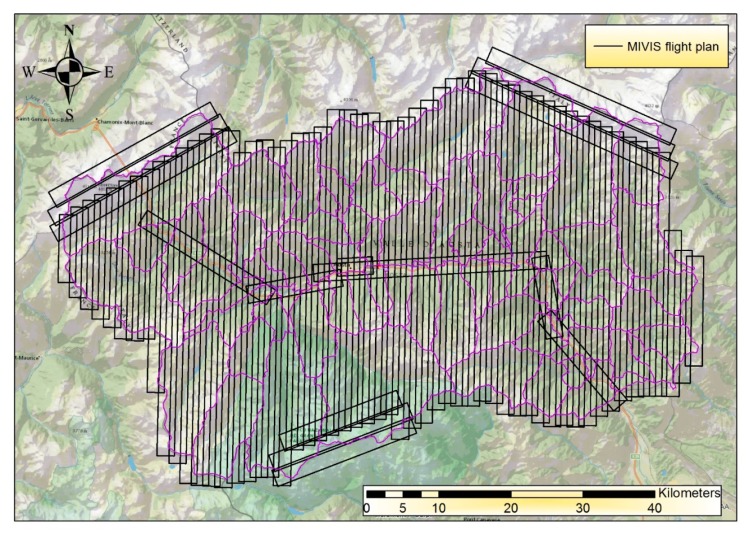
Flight plan of the aerial survey over the of the Valle d'Aosta Autonomous Region.

The data used in this study were collected with the MIVIS sensor, an airborne hyperspectral scanner featuring 102 spectral bands from visible to thermal infrared with a 2 milliradians instantaneous field of view [13]. Table 1 shows a summary of its main characteristics. At the beginning of Fall 1999, the RAVA territory was surveyed with MIVIS. The flight plan included seventy transect strips of which sixty one were used (Figure 2):

1. One block of six transect strips was acquired over the central valley, characterized by rather small changes in scale factor with a nominal spatial resolution of 4 m. This survey was used for the analysis of the main valley;
2. One block of fifty-five transect strips was acquired in the North-South direction. Since a wide range of elevations characterizes the region, these data had rather large changes in scale factor. Concerning the main urbanized areas, the nominal spatial resolution ranged from 6 m to 9 m. This survey was used for the analysis of the rest of RAVA.

In addition, ancillary data such as high resolution ortho-photos (0.5 m spatial resolution), list of roofs reclaimed from 1999 to 2011 made available by the local healthcare company (ASL), municipal boundaries, regional technical maps (1:10,000 scale) and a digital elevation model (DEM) with 10 m cell size, were available for the study.

### 2.3. Field Surveys

A first field survey for calibration purposes was carried out during winter 2007 in the valley bottom and thirty asbestos-cement roofs were surveyed. Based on the remote sensing analysis, a second field survey was carried out for validation from January 2010 to September 2011 by the technical staff of the Regional Environmental Protection Agency (ARPA Valle d'Aosta). At first, only asbestos-cement roofs detected by the classification were surveyed. In a second step, the technical staff of ARPA Valle d'Aosta surveyed other buildings (not classified as containing asbestos), resulting in 917 new asbestos-cement roofs omitted in the thematic classification.

### 2.4. Image Processing

Sixty one 102-channel MIVIS flight lines were processed. According to previous studies [15,21,22], the MIVIS data were spectrally reduced to a subset after removing the zero-value data and most of the noisy bands by means of visual inspection. The TIR data (8.18 to 12.70 μm) were excluded as well [13]. The entire data set was georeferenced to a universal transverse Mercator (Zone 32N) with ground control points collimated on the ortho-photos and orthorectified with the DEM, using the nearest neighbor interpolation algorithm to avoid changes in the radiometric values.

The corrected images were used as input to the Minimum Noise Fraction (MNF) transformation, both for the normalization of data and for reducing the residual noise [22,23]. On the basis of the eigenvalues and the spatial information contained in the output MNF transformed images, the first 7 MNF components were retained for the further analysis. The MNF technique was selected according to several recent studies which highlighted the better performances of MNF transformed data over Principal Component Analysis and Independent Component Analysis in feature extraction [24,25], target detection [26] and thematic classification [27].

The mapping of asbestos-cement roofs was performed using the Spectral Angle Mapper (SAM) algorithm. Ten asbestos-cement covers identified in the valley bottom during the first field survey were used as endmembers for thematic classification of a MIVIS subset. The selection of the classification parameters was tuned using a trial and error approach on the basis of twenty testing samples of asbestos-cement covers mapped during the same campaign [28]. Then, the image classification was applied the whole RAVA data set without any additional tuning.

## 3. Results and Discussion

**Figure 3.**
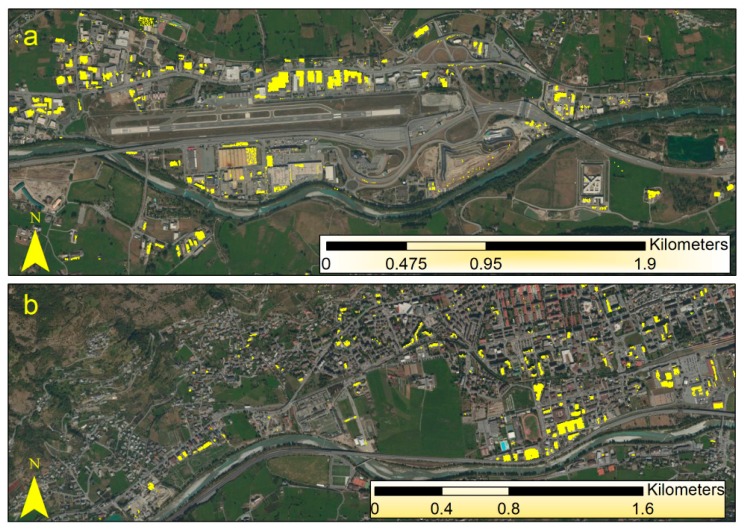
Examples of asbestos-cement roofs detection (yellow polygons) overlaid on the ortho-photos. (**a**) Commercial area near Aosta; (**b**) South-west side of Aosta.

**Figure 4.**
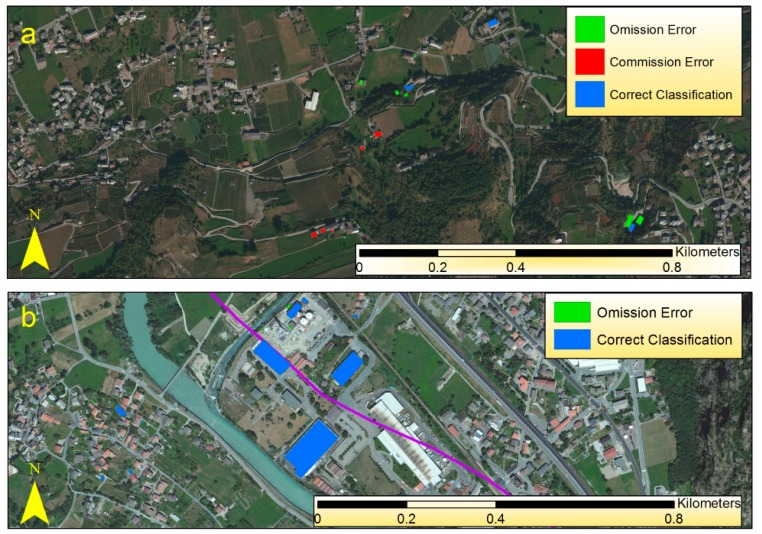
Examples of asbestos-cement roofs correct detection (blue polygons), commission error (red polygons) and omission error (green polygons). (**a**) Residential area between Gressan and Charvensod; (**b**) Industrial area between Verres and Issogne.

**Figure 5.**
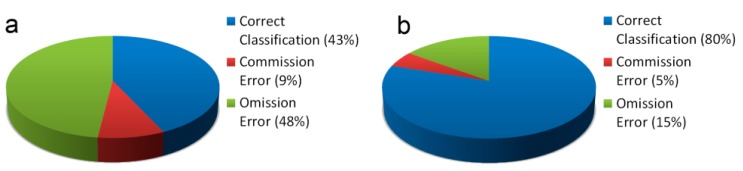
Overall classification results for the whole study area: (**a**) number of asbestos-cement roofs; (**b**) percentage of surface (m^2^) of asbestos-cement roofs.

Figure 3 shows some examples of the classification result for Aosta and Figure 4 shows some examples of correct detection and misclassification for the areas of Gressan, Charvensod, Verres and Issogne. Figure 5 and Table 2 summarize the validation results for the whole study area. Validation showed the disappearance of some asbestos-cement covers detected with MIVIS. Some buildings belonged to those reclaimed and some other buildings belonged to those involved in the flood of 2000, thus not still present during the validation campaign.

### 3.1. Classification Accuracy

With respect to asbestos-cement roof units, validation showed a large omission error (OE) but a small commission error (CE): almost half of the ground surveyed roofs were not detected with the MIVIS survey (false negatives), thus omitted in the thematic map, while less than 10% were misclassified (false positives) and representing error of commission. While some of the CE may be related to roofs reclaimed from 1999 to 2011 but not included in the ASL list, OE could be mainly explained in terms of the geometric resolution of the data set. In fact, looking at the statistics computed in terms of asbestos-cement roof area (Figure 5b, Table 2) the overall correct classification (CC) increased to 80% and the OE decreased to 15%. In other words, 80% of the asbestos-cement roofing were correctly detected from aircraft but most of the smaller covers were omitted. Thus, this method tends to underestimate the presence of asbestos-containing roofs.

**Figure 6.**
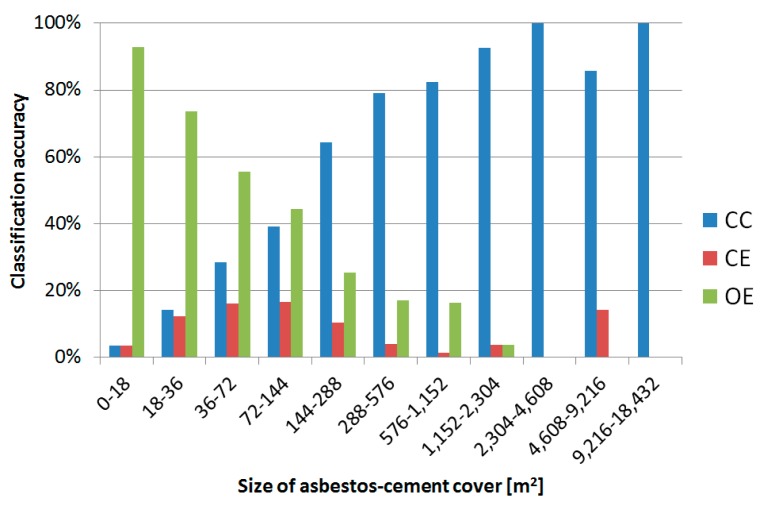
Correct detection and classification errors *vs*. the size of the asbestos-cement roofs. CC: correct classification, OE: omission error, CE: commission error.

Figure 6 shows the same phenomenon more in details. The graph clearly highlights the increase of correct detection rates and the decrease of classification errors with the increase of the size of the asbestos-cement roofs. If we consider the valley bottom, where the MIVIS data has a nominal spatial resolution of 4 m, and assume that at least a window of 3 × 3 pixel containing asbestos is needed for a correct detection (no unmixing techniques were used in this study), then only roof size larger than 144 m^2^ (RSLT144) will be classified with a reasonable confidence. This outcome is consistent with similar recent results reported by independent authors [29], as discussed later on. Regarding the RAVA case study, statistics for RSLT144, computed in terms of number of asbestos-cement roof units, improved significantly with respect to statistics for the whole data set: CC raised from 43% to 75%, while CE and OE dropped from 9% to 6% and from 48% to 19%, respectively.

### 3.2. Influence of Topographic Features

**Figure 7.**
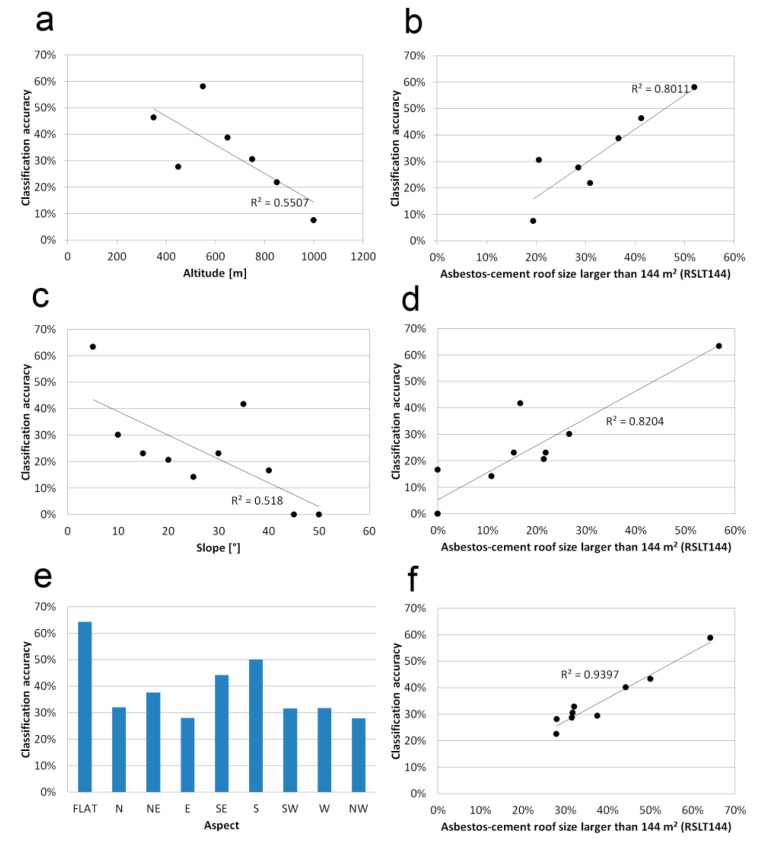
Correlation between classification accuracy and topographic features: (**a**) classification accuracy *vs*. altitude; (**b**) classification accuracy *vs*. percentage of asbestos-cement roofs larger than 144 m^2^ (same data used in plot 7a); (**c**) classification accuracy *vs.* slope; (**d**) classification accuracy *vs*. percentage of asbestos-cement roofs larger than 144 m^2^ (same data used in plot 7c); (**e**) classification accuracy *vs*. aspect; (**f**) classification accuracy *vs*. percentage of asbestos-cement roofs larger than 144 m^2^ (same data used in plot 7e).

Since the study area is a mountainous region, the influence of elevation, slope and aspect was analyzed to explain any effect of topography on the classification accuracy. Regarding the altitude, the elevation range where asbestos-cement roofs were found (from 300 m to 1900 m) was divided into 10 classes. Focusing the analysis on the first seven classes (ranging from 300 m to 1100 m), which account for more than 90% of both roof units and roof area, statistics show a weak correlation, with a R^2^ value of 0.55 (Figure 7a). However, when the percentage of RSLT144 in each altitude class is plotted against the classification accuracy, then a stronger correlation (R^2^ = 0.80) is found (Figure 7b). This suggest that the influence of the altitude on the classification performance is more related to the presence of RSLT144 (mainly located in the lower altitude classes) than the altitude itself, since the greater is the percentage of RSLT144, the greater is the classification accuracy.

Considering the slope, the range from 0° to 50° was divided into 10 classes of 5° each. As shown in Figure 7c, the slope influence presents a weak correlation (R^2^ = 0.52) on the classification accuracy, while focusing on the percentage of RSLT144 for each slope class, a stronger correlation (R^2^ = 0.82) is found (Figure 7d). As for the altitude analysis, the influence of the slope on the classification performance seems more related to the presence of RSLT144, which are mainly located in flat areas rather than in steep slopes.

Regarding the aspect, the range was divided into 8 classes (*i.e.*, North, North-East, East, South-East, South, South-West, West and North West) with the addition of the class “Flat” (*i.e.*, no aspect). In this case, the highest classification's accuracies were obtained for the classes “Flat” (64%) and “South” (50%), as shown in Figure 7e. These results can be explained again in terms of percentage of RSLT144 in each aspect class. In fact, flat areas and South-facing slopes contained much more large asbestos-cement roofs than the other aspects. Thus, a strong correlation (R^2^ = 0.94) was found between the percentage of RSLT144 of aspect classes and the classification accuracy (Figure 7f).

### 3.3. Comparison to Past Studies

With respect to past studies using hyperspectral remote sensing, the mapping accuracy calculated for RAVA case history is comparable to those achieved by other authors. Bassani *et al.* studied two transect strips: one (about 8.5 km^2^) collected over the industrial area of Follonica (Italy) and another (about 1 km^2^) collected over Rimini (Italy), reporting a correct identification of asbestos-cement roofs ranging from 80% to 90% [13]. Cavalli *et al.* studied three transect strips (overall about 123 km^2^) collected over Podgorica (Montenegro), reporting a mapping accuracy ranging from 80% to 90% [30]. Basile Giannini *et al.* studied a transect strip (about 16 km^2^) collected over Reggio Calabria (Italy), reporting an accuracy of 83.5% in the mapping of asbestos-cement roofs using a method similar to that used in this study [16]. Fiumi *et al.* studied a transect strips (about 5 km^2^) collected over Roma (Italy), reporting an extraordinary accuracy of about 94% for asbestos-cement [18]. In this case, however, it should be noted that almost all the buildings identified as containing asbestos-cement material (n = 32) with the MIVIS survey had a quite large roof surface (average area of about 1200 m^2^) [18].

All these studies, however, mainly focused on the methodological aspects and the data processing was often limited to small test sites or patches, typically in flat areas or with gently undulating terrain. Some exceptions are the recent studies of Sciunnach *et al.* and Fiumi *et al*., where the mapping of asbestos-cement roofing was performed over large areas, in a real operational scenario. In the first study, about 9% of Lombardia (Northern Italy) was surveyed with MIVIS, corresponding to a surface of 2000 km^2^ [19]. The authors claimed a mapping accuracy of about 90%, but no further details were given, so a comparison of results with the RAVA case study is not possible. Anyway, in literature this seems the most similar case history of asbestos-cement mapping over a large urbanized area using airborne remote sensing techniques. The second study investigated about 4.6% of Lazio (Central Italy), corresponding to a surface of 800 km^2^. This study area is less than a half of that analysed in Lombardia and only a fourth of that analysed in RAVA. The authors reported a mapping accuracy of asbestos-cement coverage ranging from 67% to 75%, based on testing samples larger than 150 m^2^ (16 image pixels), very similar to our results [17].

Regarding mountainous areas with steep slopes and great elevation range such as the RAVA, no relevant case histories have been described in literature. Mountain areas can introduce some specific issues, such as shadows, which may bias the mapping [21]. This study demonstrated that the mountainous topography does not significantly affects the accuracy of the method proposed, making it suitable for surveys in other similar locations.

At present, limitations in the use of more affordable cost satellite data are mostly due to inadequate spectral and/or spatial resolution. Some examples are reported by Armesto González *et al.* and Hyun and Park. The first authors described an attempt to map asbestos materials using Ikonos images but, despite the high spatial resolution (4 m), its poor spectral resolution (4 broad bands in the visible and near-infrared spectrum) allowed only a rough classification of buildings with either metal or asbestos cement roofs [31]. The second study tested Hyperion data for mapping asbestos roofing. In this case, despite the high spectral resolution (220 continuous narrow bands covering the spectral range from 0.4 μm to 2.5 μm), its insufficient spatial resolution (30 m) led to a poor accuracy ranging from 30% to 60% [20].

## 4. Conclusions

This study demonstrated how airborne hyperspectral data could be useful for supporting the mapping of asbestos-cement roofs on a very large mountainous area, thus in real operational scenario of the Alps. Past studies mainly focused on the methodological aspects and often analyzed small geographic extensions, typically in flat areas or with gently undulating terrain, while no relevant literature is available for mountainous areas. The large extension of the study area is an issue especially for airborne surveys that need many flight lines imaged in different days and time. Moreover, data may have different spatial resolution and different atmospheric and illumination conditions, making difficult the processing of the whole data set.

The major findings of this research are related to the roof's size and to the terrain's topography. Regarding the size of asbestos-cement covers, validation pointed out that 3 × 3 image pixels is a threshold for a good detection. When considering all the real asbestos-cement roofs, regardless their size, the mapping with MIVIS data correctly recognized only 43%. On the other hand, when considering asbestos-cement roofs larger than 3 × 3 image pixels, the correct detection was 75%. If looking at the roofs' surface, the overall accuracy increased to 80%. These results are consistent with the outcomes of past studies and are confirmed also for mountainous areas.

Regarding the topography, the on-site survey of the whole territory carried on after the analysis demonstrated that in a mountainous area topographic features do not significantly affects the accuracy of the mapping, making it suitable for surveys in other similar locations. Even if some weak correlations between the mapping accuracy and the topographic features (*i.e.*, elevation, slope and aspect) were found, they seem to be more related to the presence of larger asbestos-cement roofing rather than to the topographic features themselves. This result seems to suggest that even in high relief landscapes the spatial resolution of data is the major source of errors.

A peculiarity of this research is its validation. Usually, map validation is based on some (few) spots surveyed but here all the buildings in RAVA were checked for accessing the reliability of the methodology. This means that the validation statistics were calculated on all the existing asbestos-cement roofs, thus correct classification and error rates were not extrapolated form a testing subset.

In conclusion, regardless the temporal difference between the flight, data processing and field surveys (definitely a weakness of the study which complicated the analysis), the remote sensing analysis provided useful information to help the authorities in mapping the asbestos-cement roofing on a very large and complex area using a simple data processing workflow and few endmembers.

## Figures and Tables

**Table 1.** Summary of the MIVIS characteristics (1999).

Sensor Bands	102
**Spectral Range**	VIS: 430–830 nm
	NIR: 1150–1550 nm
	SWIR: 2000–2500 nm
	TIR: 8200–12,700 nm

**Spectral Resolution**	VIS: 20 nm
	NIR: 50 nm
	SWIR: 8 nm
	TIR: 400–500 nm

**Instantaneous Field of View**	2 mrad

**Swath Width**	755 pixel

**Dynamic Range**	12-bits per pixel

**Scan Speed**	8.3, 12.5, 16.7, 25 Hz

**Table 2.** Summary of results.

	Correct Classification	Commission Error	Omission Error	Total
Roof units [nr.]	833	181	928	1942
Total surface [m^2^]	431,266	28,398	82,766	542,429

## References

[1] Bartrip P.W.J. (2004). History of asbestos related disease. Postgrad. Med. J..

[2] Reid A., de Klerk N.H., Magnani C., Ferrante D., Berry G., Musk A.W., Merler E. (2014). Mesothelioma risk after 40 years since first exposure to asbestos: A pooled analysis. Torax.

[3] Luqman M., Javed M.M., Daud S., Raheem N., Ahmad J., Khan A.U.H. (2014). Risk factors for lung cancer in the Pakistani population. Asian Pac. J. Cancer Prev..

[4] Offermans N.S.M., Vermeulen R., Burdorf A., Goldbohm R.A., Kauppinen T., Kromhout H., van den Brandt P.A. (2014). Occupational asbestos exposure and risk of pleural mesothelioma, lung cancer, and laryngeal cancer in the prospective Netherlands cohort study. J. Occup. Environ. Med..

[5] Camargo M.C., Stayner L.T., Straif K., Reina M., Al-Alem U., Demers P.A., Landrigan P.J. (2011). Occupational exposure to asbestos and ovarian cancer: A meta-analysis. Environ. Health Perspect..

[6] Wang X., Courtice M.N., Lin S. (2014). Cumulative incidence of asbestosis and exposure levels in a chinese asbestos worker cohort. Am. J. Respir. Crit. Care Med..

[7] World Health Organization Asbestos: Elimination of Asbestos-related Diseases. Fact Sheet N.343 2014. http://www.who.int/mediacentre/factsheets/fs343/en/.

[8] Toxic Substances Control Act (TSCA) US Environmental Protection Agency. http://www.epa.gov/oecaagct/lsca.html.

[9] (1976). Council Directive 76/769/EEC of 27 July 1976 on the approximation of the laws, regulations and administrative provisions of the Member States relating to restrictions on the marketing and use of certain dangerous substances and preparations (76/769/EEC). Off. J. Eur. Commun..

[10] Commission Directive 99/77/EEC. http://www.chem-envi.com/info/1999-77-EC.pdf.

[11] (1992). Legge 27 marzo 1992, n. 257—Norme relative alla cessazione dell'impiego dell'amianto. Suppl. Ordin. Alla Gazz. Uff..

[12] (2003). Decreto 18 marzo 2003, n. 101—Regolamento per la realizzazione di una mappatura delle zone del territorio nazionale interessate dalla presenza di amianto, ai sensi dell'articolo 20 della legge 23 marzo 2001, n. 93. Gazz. Uff..

[13] Bassani C., Cavalli R.M., Cavalcante F., Cuomo V., Palombo A., Pascucci S., Pignatti S. (2007). Deterioration status of asbestos-cement roofing sheets assessed by analyzing hyperspectral data. Remote Sens. Environ..

[14] Marino C., Panigada C., Busetto L. (2001). Airborne hyperspectral remote sensing applications in urban areas: Asbestos concrete sheeting identification and mapping.

[15] Fiumi L. (2001). Evaluation of MIVIS hyperspectral data for mapping covering materials.

[16] Basile Giannini M., Creta T., Guglietta D., Merola P., Allegrini A. (2012). Methodologies to identify asbestos-cement roofing by remote data. Ital. J. Remote Sens..

[17] Fiumi L., Congedo L., Meoni C. (2014). Developing expeditious methodology for mapping asbestos-cement roof coverings over the territory of Lazio Region. Appl. Geomat..

[18] Fiumi L., Campopiano A., Casciardi S., Ramires D. (2012). Method validation for the identification of asbestos-cement roofing. Appl. Geomat..

[19] Sciunnach D., Bellingeri D., Capetta C., Cappa S., Cornaggia N., Losa L., Mensi C., Somigliana A., Zini E. Asbestos disposal in Lombardy (Northern Italy).

[20] Hyun C.-U., Park H.-D. (2010). Hyperspectral remote sensing of serpentine rocks and asbestos bearing roofing slate.

[21] Shahtahmassebi A., Yang N., Wang K., Moore N., Shen Z. (2013). Review of shadow detection and de-shadowing methods in remote sensing. Chin. Geogr. Sci..

[22] Frassy F., Morra Di Cella U., Bocca M., Bovio M. Habitat cartography using color infrared and hyperspectral images.

[23] Frassy F., Dalla Via G., Maianti P., Marchesi A., Rota Nodari F., Gianinetto M. Minimum noise fraction transform for improving the classification of airborne hyperspectral data: Two case studies.

[24] Amato U., Cavalli R.M., Palombo A., Pignatti S., Santini F. (2009). Experimental approach to the selection of the components in the minimum noise fraction. Trans. Geosci. Remote Sens..

[25] Dópido I., Zortea M., Villa A., Plaza A., Gamba P. (2011). Unmixing prior to supervised classification of remotely sensed hyperspectral images. IEEE Geosci. Remote Sens. Lett..

[26] Galal A., Hassan H., Imam I.F. (2012). A novel approach for measuring hyperspectral similarity. Appl. Soft Comput..

[27] Du Q., Fowler J.E., Ma B. Random-projection-based dimensionality reduction and decision fusion for hyperspectral target detection.

[28] Gianinetto M., Villa P. (2007). Rapid response flood assessment using minimun noise fraction and composed spline interpolation. IEEE Trans. Geosci. Remote Sens..

[29] Frassy F., Candiani G., Maianti P., Marchesi A., Rota Nodari F., Rusmini M., Albonico C., Gianinetto M. Airborne remote sensing for mapping asbestos roofs in Aosta Valley.

[30] Cavalli R.M., Pasucci S., Pignatti S. Hyperspectral remote sensing data to map hazardous materials in a rural and industrial district: The Podgorica dwellings case studies.

[31] Armesto Gonzalez J., Gil Docampo M.L., Canas Guerrero I. (2006). The application of newtechnologies in construction: Inventory and characterisation of rural constructions using the Ikonos satellite image. Build. Environ..

